# The ubiquitin ligase NKLAM promotes apoptosis and suppression of cell growth

**DOI:** 10.1016/j.jbc.2025.108527

**Published:** 2025-04-22

**Authors:** Paul A. Willard, Jacki Kornbluth

**Affiliations:** 1 Department of Pathology, Saint Louis University School of Medicine, St Louis, Missouri, USA; 2 Research and Development Service, St Louis VA Medical Center, St Louis, Missouri, USA

**Keywords:** apoptosis, c-Myc, cell death, cell metabolism, E3 ubiquitin ligase, natural killer cells, NKLAM, RBR ubiquitin ligase, RNF19b

## Abstract

Natural killer lytic-associated molecule (NKLAM), also known as RNF19b, is a member of the RING-in between-RING-RING (RBR) E3 ubiquitin ligase family and plays a pivotal role in immune regulation. We identified a critical cysteine residue at position 301 essential for NKLAM's ubiquitin ligase function. Site-directed mutagenesis of this residue to serine or alanine abrogated the ligase activity of NKLAM. Utilizing inducible expression systems in two different cell lines, HEK293 embryonic kidney cells and K562 myeloid leukemia cells, we demonstrated that wild-type (WT) NKLAM, but not the catalytically inactive NKLAM alanine mutant (C301A), inhibited cellular proliferation, as evidenced by reduced cell numbers and decreased metabolic activity. Moreover, NKLAM expression led to a significant decrease in the abundance and stability of the proto-oncogene c-Myc, a key regulator of proliferation. NKLAM facilitated the proteasomal degradation of c-Myc, with a reduction in c-Myc half-life from 27 min to 12 min and restoration of c-Myc levels upon proteasome inhibition. Notably, prolonged NKLAM expression induced apoptosis, measured by annexin-V staining and caspase activation. Strikingly, the serine mutant, C301S, while lacking ubiquitin ligase activity, induced apoptosis comparable to WT NKLAM, highlighting an alternative pathway for NKLAM-mediated inhibition of cellular homeostasis. Our findings indicate that NKLAM is a cytolytic protein with multifaceted roles in cellular proliferation and apoptosis.

Natural killer (NK) cells are the first line of defense in response to a variety of diseases and infections. The effector function of NK cells is tightly regulated to maintain the balance of removing unhealthy cells while preventing damage to normal tissue. Signaling through activating and inhibitory receptors modulates NK cell activation thresholds, ensuring discrimination between healthy and abnormal cells. The NK cell’s ability to identify and kill stressed, infected, or transformed cells without prior sensitization makes it a formidable defensive agent. NK cells have potent effector function, using a twofold process in which the target cell receives membrane signaling to initiate extrinsic apoptosis pathways (1, 2), and takes up cytolytic molecules released by granule exocytosis that disrupt cellular homeostasis and directly activate apoptosis (3, 4).

Upon encountering target cells, NK cells form immunological synapses (5). Death receptors at the plasma membrane of the target cell are activated, stimulating the formation of the death-inducing signaling complex, resulting in the activation of initiator caspases 8 and 10 (6, 7, 8). At the same time, NK cells polarize their lytic granules toward the immune synapse, where the granules fuse with the plasma membrane, facilitating the focused delivery of cytotoxic payloads. Perforin, a pore-forming protein, perforates the target cell membrane, enabling the entry of apoptosis-inducing molecules like granzymes and granulysin (9). Granzymes, a family of serine proteases, cleave key cellular substrates, triggering apoptosis and effectively dismantling the target cell (10, 11). Furthermore, granulysin causes permeability of the lysosome membrane and release of lysosomal proteases, contributing to the destruction of the target cell (12, 13, 14).

Natural killer lytic-associated molecule (NKLAM) was discovered in a differential screening of genes upregulated in cytokine-stimulated NK cells and cytotoxic T lymphocytes (15). It is expressed at low levels in unstimulated immune effector cells, but expression rapidly increases with stimulation by IL-2, interferon (IFN)-β, or other activating cytokines (16). NKLAM is a transmembrane protein that colocalizes with granzymes in cytolytic granules and is found on extracellular vesicles secreted by NK cells (16, 17). Knockdown of NKLAM in NK cells results in diminished effector function against tumor cells, while NKLAM-deficient (NKLAM^−/−^) mice have an impaired ability to limit tumor growth and metastasis (18). NK cells isolated from NKLAM^−/−^ mice have decreased cytotoxic activity and decreased cytokine production (15, 16). Similarly, NKLAM^−/−^ macrophages have decreased efficacy in bacterial killing and cytokine production (19, 20, 21). NKLAM’s role in immune functions is broad and is the subject of continued investigations.

NKLAM, also designated RNF19b, is a membrane-bound E3 ubiquitin ligase of the RING-in between-RING-RING (RBR) family. Each member of this small family of ubiquitin ligases has an RBR ligase domain, comprised of three really interesting new gene (RING) domains referred to as RING1, the in-between RING (IBR), and RING2 (sometimes called Rcat, or catalytic RING) (22). RING domains are zinc-coordinating structures with a “finger” section that presents a surface for interactions with other molecules (23). RBR ligases contain a conserved catalytic cysteine in RING2 that enables RBR ligases to form an intermediate E3-ubiquitin conjugate prior to ubiquitin ligation to a substrate protein.

Ubiquitin (Ub) is a small protein used as a post-translational modification. Ubiquitination of a protein can have many effects, including altering enzymatic activity, changing protein localization, generating structural rearrangements, or inducing degradation (24, 25). Ubiquitins are attached to proteins in a 3-step process that involves activation by an E1 activating enzyme, transfer of Ub to an E2 conjugating enzyme, and ligation to a protein by an E3 ligase. The final step results in ubiquitination of a lysine on a substrate protein or attachment of Ub to another Ub to form a polyubiquitin chain (26).

The discovery that NKLAM expression is upregulated when cytolytic activity is enhanced and is colocalized with other cytolytic proteins in NK granules and extracellular vesicles predicts that it is similarly delivered to target cells, where it may have cytolytic activity. Efforts to establish stable transfected cell lines with constitutive NKLAM expression have been unsuccessful, further supporting the hypothesis that NKLAM is cytotoxic.

In this report, we examined the role of NKLAM in an ectopic expression model to understand what impact it has on cellular homeostasis. K562 and HEK293 cell lines were stably transfected with NKLAM or ligase-deficient NKLAM mutant constructs under the control of a doxycycline (Dox)-inducible promoter. K562 is a myeloid leukemia cell line and well characterized model target of NK effector function. HEK293 cells are representative of transformed cells that are largely resistant to NK-mediated lysis.

## Results

### Mutation of C301 abrogates the ubiquitin ligase activity of NKLAM

**Figure 1:**
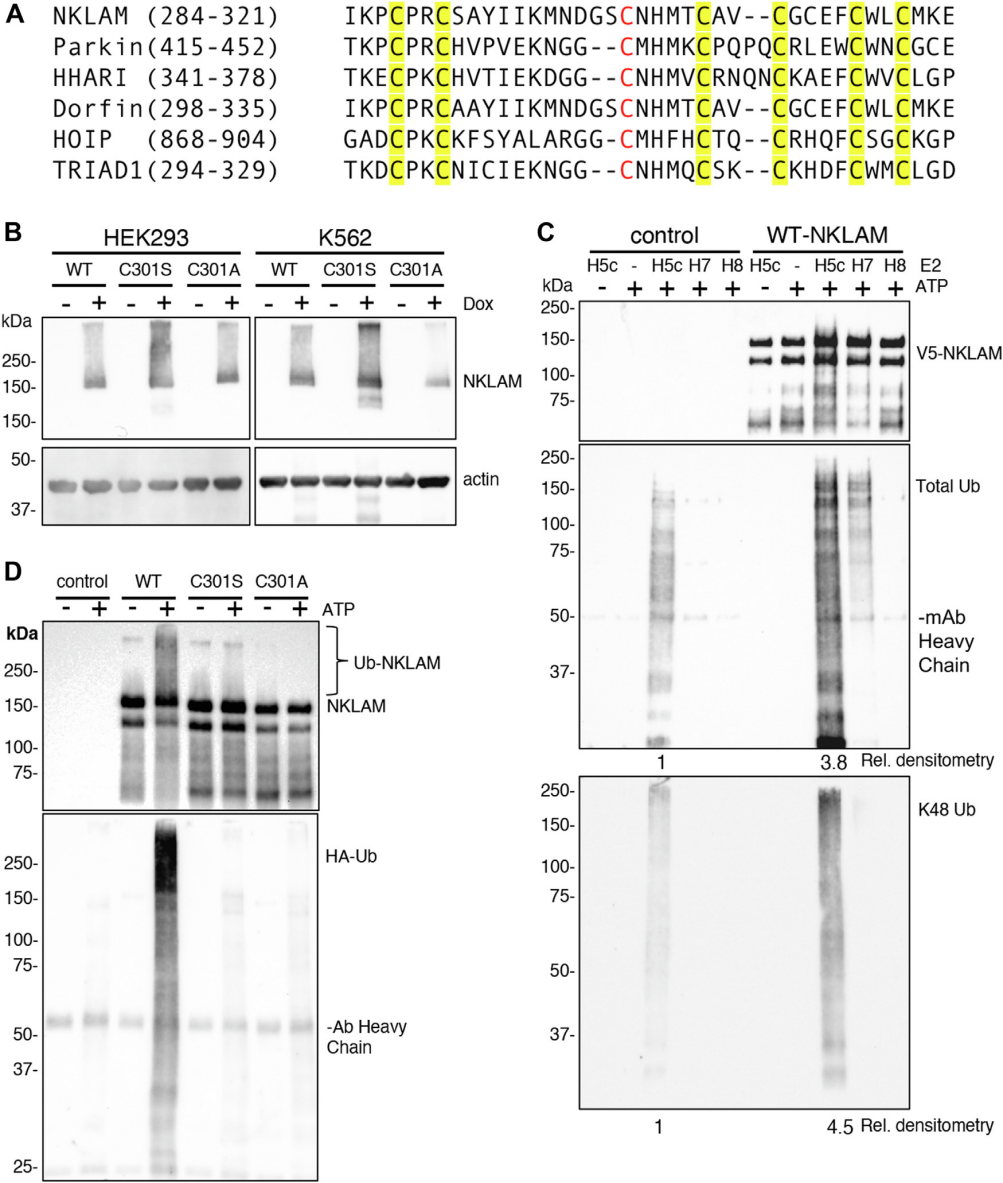
**Mutation of C301 eliminates NKLAM Ub ligase activity.***A*, sequence alignment of RING2 sequences from select RBR E3 Ub ligases with the catalytic cysteine in *red* and zinc-coordinating cysteines in yellow. *B*, immunoblots of NKLAM from lysates of HEK293 and K562 cells treated overnight with DMSO (−) or Dox (+). Actin serves as a loading control. *C*, *in vitro* Ub ligase assays with NKLAM IP’d from non-induced or WT NKLAM-expressing HEK293 combined with E2 enzymes UbcH5 (H5c), UbcH7 (H7) and UbcH8 (H8). Negative control reactions lack ATP. Ligase reactions were immunoblotted for NKLAM, total Ub (HA-Ub) and K48-Ub. Fold differences in Ub are shown below each blot. *D*, *in vitro* Ub ligase assays with WT NKLAM, C301S, and C301A proteins IP’d from Dox-induced HEK293 cells combined with UbcH7. Negative control reactions lack ATP.

All RBR ubiquitin ligases have a highly conserved cysteine in the RING2 domain. This was experimentally determined to be the catalytic cysteine in several RBR ligases. Sequence alignment of RING2 from these RBR ligases with RING2 of NKLAM is shown in Figure 1*A*. The sequence alignment and constructs of NKLAM utilized in these studies are based on isoform 4 (UniProt ID: Q6ZMZ0-4). This isoform of NKLAM was the first to be discovered and differs from isoform one by a single alanine deletion at position 281, which occurs at an RNA splice site. Using site-directed mutagenesis, the putative catalytic cysteine of NKLAM was mutated into either serine or alanine to create the NKLAM constructs C301S and C301A, respectively. Both mutations are predicted to eliminate ubiquitin ligase activity. The serine mutant would present a hydroxymethyl group at the catalytic site, which can form a non-reactive ester bond with Ub, effectively trapping Ub at the site. The alanine mutation would be non-reactive, unable to bind activated Ub.

The Sleeping Beauty transposon was used to generate HEK293 and K562 cells with Dox-inducible expression of wild-type (WT) NKLAM, C301S, or C301A (27, 28). All NKLAM constructs have fluorescent mScarlet and V5 epitope tags on their 3′ ends. Dox concentrations were tested and adjusted until a roughly equivalent expression of the 3 NKLAM products was achieved (Fig. 1*B*). The primary band representing NKLAM, with mScarlet and V5 attached, runs at approximately 150 kDa, despite its predicted molecular weight of 110 kDa. This observation is consistent with previously published data showing untagged endogenous NKLAM at 110 kDa, when the predicted molecular weight of NKLAM is 80 kDa (16, 29, 30).

To assess ubiquitin ligase function, NKLAM was immunoprecipitated (IP’d) using anti-V5 or anti-NKLAM antibodies onto protein G beads from control and Dox-induced HEK293 cells. We first tested NKLAM ubiquitin ligase activity with three different E2 enzymes (Fig. 1*C*). UbcH5c was selected due to its recognized 'promiscuous' activity, capable of donating active Ub to a broad spectrum of E3 ligases (31). UbcH7 and UbcH8, previously demonstrated to interact with NKLAM, were included to assess their roles as potential Ub donors for NKLAM (29). Ubiquitination by NKLAM was observed with both UbcH5c and UbcH7, and total ubiquitin smearing ran the entire length of the membrane, starting at about 25 kDa. The ligase reaction with UbcH7 was highly specific, with no background activity in non-induced control samples, but with less signal compared to UbcH5. Densitometry analyses indicated a nearly 4-fold increase in ubiquitin ligase activity for the NKLAM sample with UbcH5c compared to its non-induced V5-IP control. The background activity with the UbcH5 reaction was likely caused by one or more E3 ligases binding non-specifically to the protein G beads. Ubiquitination by NKLAM was not observed with UbcH8. K48 and K63 ubiquitin linkages were also probed in this assay; only K48 linkages could be detected in the NKLAM reaction with UbcH5c. K48 linkages were confirmed in a follow-up assay using UbcH7, ensuring that the linkages were specific to NKLAM activity (Fig. S1).

We tested the ligase activity of the C301S and C301A mutants using UbcH7 (Fig. 1*D*). No ubiquitination was seen with the mutant forms of NKLAM, indicating that the mutation of C301 renders NKLAM ligase deficient. This assay not only demonstrated the lack of ligase function by the mutants but also ensured that ligase activity seen with the WT NKLAM construct was specific to NKLAM and not due to an E3 ligase that might have copurified with NKLAM. Mutant forms of NKLAM were equally deficient in ligase activity. Probing of immunoblots for NKLAM revealed significant smearing of WT NKLAM only, indicating its possible auto-ubiquitination.

### NKLAM expression inhibits proliferation

**Figure 2:**
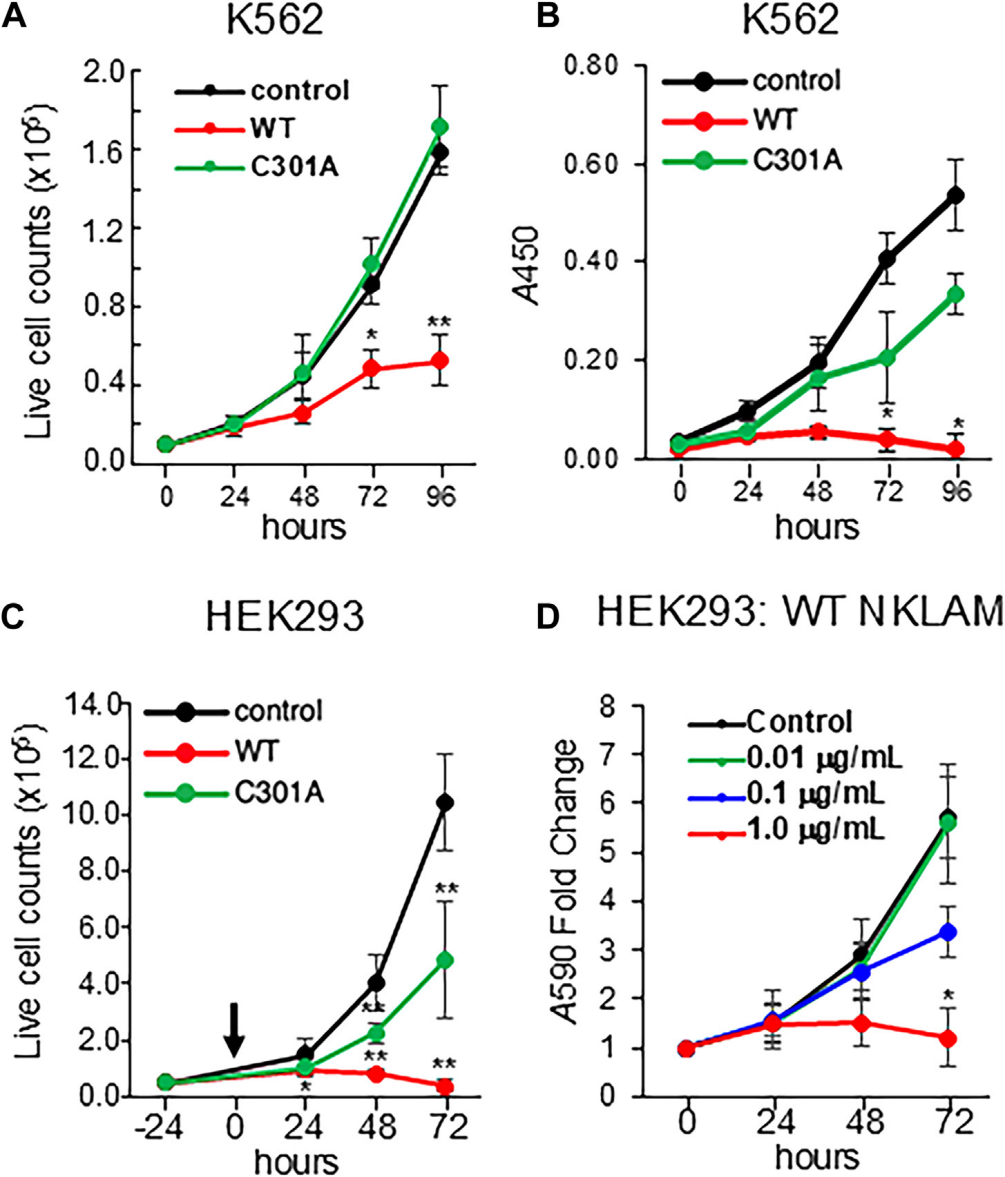
**NKLAM expression inhibits cellular proliferation.***A*, cell counts of K562 over 96 h. Plates were seeded with 5000 cells per well at time 0 and treated with either DMSO (control) or 1 μg/ml Dox to induce WT NKLAM or NKLAM C301A (n = 3 experiments, three replicates per experiment). *B*, WST-1 assays of K562 cells measured by A450 over 96 h 5000 cells were seeded per well at time 0 and incubated with WST-1 reagent for 2 h prior to measurement (n = 3 experiments, three replicates per experiment). *C*, HEK293 cell counts over 72 h of NKLAM induction. 5000 cells were seeded per well and treated with DMSO or Dox at time 0 (arrow) and counted every 24 h for 3 days (n = 3 experiments, three replicates per experiment). *D*, dose-dependent inhibition of HEK293 metabolic activity by NKLAM. Crystal violet assays of HEK293 cells treated with DMSO or a titration of Dox at 1.0, 0.1 and 0.01 μg/ml, to induce WT NKLAM expression. 2000 cells were seeded per sample and crystal violet measured every 24 h for 3 days after induction (n = 3 experiments, three replicates per experiment). ∗*p* < 0.05, ∗∗*p* < 0.0005.

After establishing NKLAM-inducible cell lines and confirming the ligase-deficient nature of the C301 mutant, we tested the effect of NKLAM on cell growth. We compared the expression of WT NKLAM and C301A on the proliferation of K562 and HEK293 cells by conducting a series of proliferation assays spanning 3 to 5 days. Non-induced cells treated with the Dox diluent DMSO served as our control group. Cells were counted at 24-h intervals after seeding. Expression of WT NKLAM inhibited proliferation (Fig. 2*A*). In contrast, K562 cells expressing C301A exhibited no difference in proliferation compared to the non-induced control cells.

To complement these observations, a WST-1 assay was performed (Fig. 2*B*), measuring mitochondrial dehydrogenase activity as an indicator of metabolic activity and an indirect measure of cell number and viability. This assay revealed a reduction in dehydrogenase activity in WT NKLAM-expressing cells, whereas cells expressing the inactive mutant showed an increase in signal over time, similar to the DMSO control. These two assays made clear the negative impact NKLAM expression has on proliferation. Not only did WT NKLAM prevent proliferation, but metabolic activity measured by WST-1 decreased over time. Expression of ligase-dead C301A had no significant impact on K562 cell growth or metabolic activity.

Expression of WT NKLAM in HEK293 cells resulted in a significant decrease in proliferation, mirroring observations in K562 cells (Fig. 2*C*). Notably, HEK293 cells expressing C301A also demonstrated reduced proliferation, albeit not to the same degree as WT NKLAM. A crystal violet assay was conducted with HEK293 cells expressing NKLAM to evaluate proliferation using an orthogonal method to cell counts (Fig. 2*D*). A Dox titration experiment revealed a dose-response relationship between proliferation and WT NKLAM expression. However, when performed with C301A-expressing HEK293 cells, the cells were less adherent to the plate, leading to inconsistent measurements. This adhesion issue may also account for the modest decrease in proliferation observed in HEK293 cells expressing C301A in Figure 2*C*.

### NKLAM expression decreases c-myc abundance and stability

**Figure 3:**
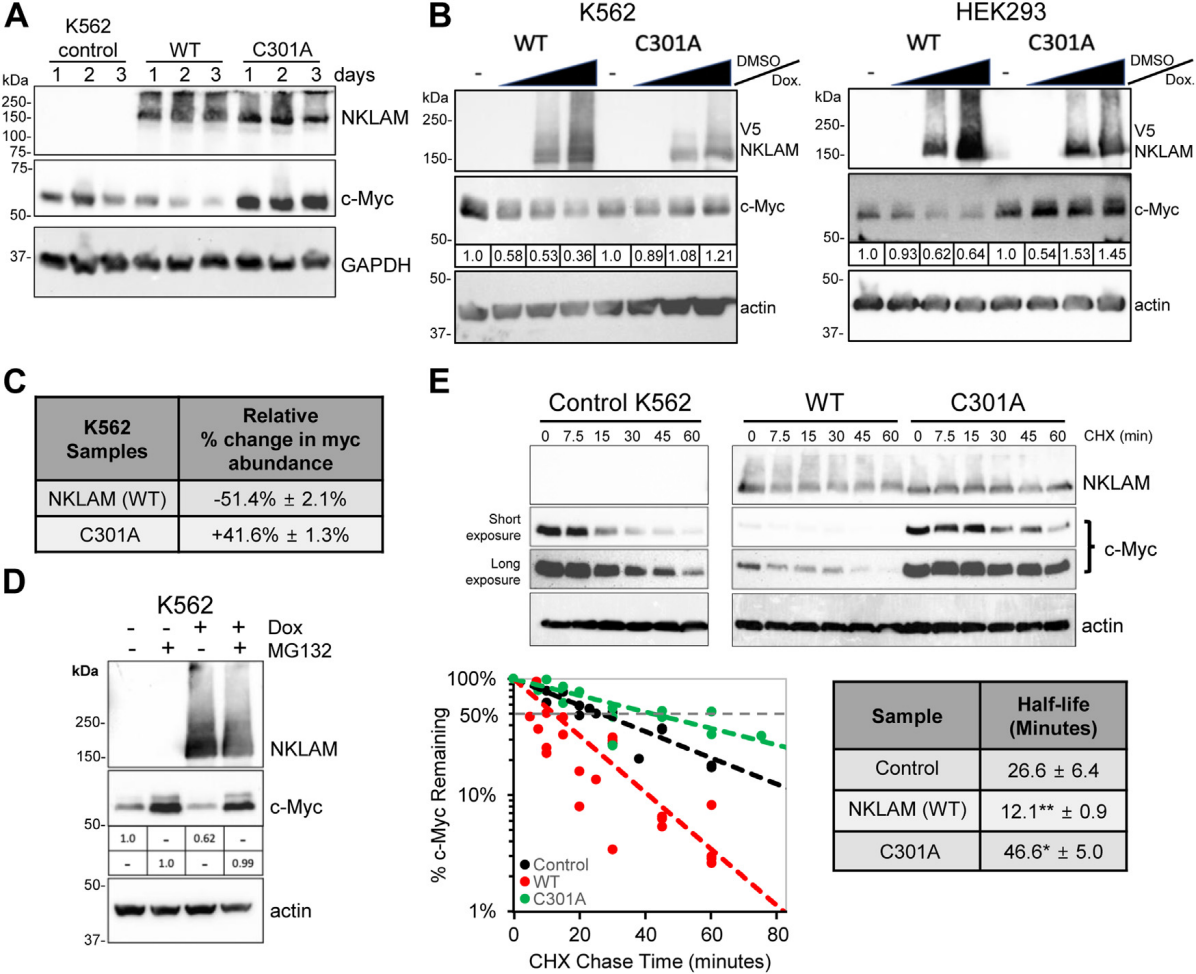
**NKLAM expression lowers c-Myc protein abundance and decreases c-Myc half-life.***A*, immunoblot of c-Myc levels in non-induced or K562 cells induced to express WT or the C301A NKLAM mutant for 1, 2 and 3 days. GAPDH serves as a loading control. *B*, dose-dependent decrease in c-Myc with increased expression of WT NKLAM. Immunoblots of DMSO control and a titration of 1.0, 0.1 and 0.01 μg/ml Dox to induce WT and C301A mutant NKLAM expression in K562 (*left*), and HEK293 cells (*right*). Actin serves as a loading control. c-Myc densitometry analyses are normalized to both actin and respective non-induced controls. *C*, densitometry of c-Myc in WT and C301A expressing K562 cells after 24 h, normalized to the non-induced control (n = 4). *D*, control and WT NKLAM-expressing K562 with and without MG132. Numbers reflect levels of c-Myc relative to their treatment matched control. *E*, representative immunoblot of a CHX assay and analysis of c-Myc half-life in K562 control cells and cells expressing NKLAM WT or the C301A mutant. Regression analysis of c-Myc half-life and table of results averaged from four independent experiments. ∗*p* = 0.002, ∗∗*p* = 0.0006.

Considering c-Myc's involvement in cellular proliferation and the observed inhibition of proliferation associated with NKLAM expression, our investigation focused on exploring the potential impact of NKLAM on c-Myc abundance. Immunoblot analyses of cell lysates collected over a 72-h period demonstrated a progressive reduction in c-Myc abundance in cells expressing NKLAM (Fig. 3*A*). Moreover, this decrease was NKLAM dose-dependent, evidenced by the titration of Dox for both K562 and HEK293 cells (Fig. 3*B*). The decrease in c-Myc was not observed in C301A-expressing cells; rather, C301A-expressing cells showed an increase in c-Myc abundance. The mean change in c-Myc abundance observed was a 51.4% ± 2.1% decrease in WT NKLAM-expressing cells and a 41.4% ± 1.3% increase in c-Myc abundance in cells expressing C301A (Fig. 3*C*).

It is well documented that c-Myc is degraded in a proteasome-dependent manner (32), so we investigated this process in the presence of NKLAM. NKLAM expression was induced for 24 h in K562 cells. Subsequently, the control and induced cultures were divided and treated with either DMSO or the proteasome inhibitor MG132 for 3 h (Fig. 3*D*). Following treatment, cultures were harvested, lysed, and subjected to immunoblot analysis. MG132 treatment of control cultures increased c-Myc abundance by 4.6-fold. NKLAM-expressing cells initially had 40% less c-Myc than control cells; MG132 treatment significantly increased the abundance of c-Myc in NKLAM-expressing cells by 7.3-fold, bringing the total abundance to parity with the MG132-treated non-induced control cells.

To further investigate the impact of NKLAM on c-Myc degradation, a cycloheximide (CHX) assay was employed to monitor changes in c-Myc abundance over time and measure its half-life (Fig. 3*E*). The half-life of c-Myc was determined by treating cells with the protein synthesis inhibitor CHX and then following the loss of the existing protein over time. Typically, c-Myc has a half-life of about 30 min, although this duration may vary among cell lines (33). In our analysis, the half-life of c-Myc in K562 cells was 27 min. However, upon expression of WT NKLAM, the half-life significantly decreased to less than half, measuring at 12 min. Interestingly, expression of C301A appeared to increase c-Myc stability, raising the half-life to 47 min.

Experiments were conducted to determine the ubiquitination state of c-Myc by various methods (Fig. S2). To probe for c-Myc ubiquitination, cells were incubated with DMSO or Dox overnight, then treated with MG132 for 3 h. Cells were lysed and lysates were incubated with tandem ubiquitin binding entities (TUBEs) to pull down ubiquitinated proteins (34). TUBEs pull-down products were immunoblotted for c-Myc. Using this assay, we could detect ubiquitinated c-Myc in control cells but observed no increase in ubiquitination of c-Myc in the presence of NKLAM. We also could not detect ubiquitinated c-Myc in c-Myc pulldown experiments (data not shown).

### NKLAM induces apoptosis

**Figure 4:**
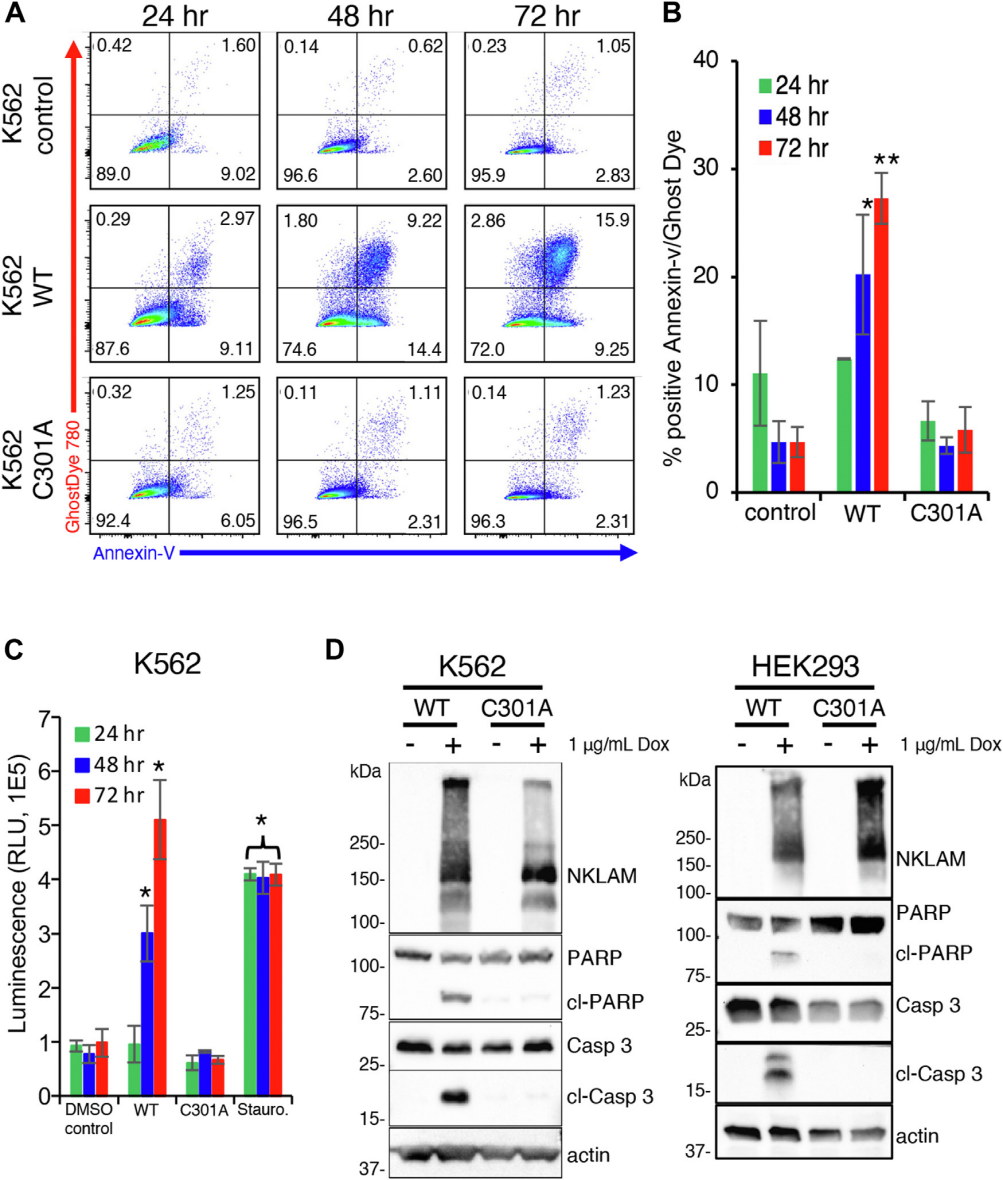
**NKLAM expression induces caspase activation and apoptosis.***A*, representative flow cytometry scatter plots of annexin-V and Ghost Dye staining of non-induced K562 control, WT NKLAM or C301A expressing cells at 24, 48 and 72 h. *B*, percentage of cells positive for annexin-V, Ghost Dye, or both from flow cytometry assays (n = 3 separate experiments). ∗*p* < 0.02, ∗∗*p* < 0.01. *C*, caspase-Glo 3/7 luminescence assay of K562 cells with DMSO or Dox-induced expression of WT or C301A NKLAM. Relative light units (RLU) reflect the levels of activated caspases three and 7. Staurosporine (Stauro) is a positive control for apoptosis (n = 3 separate experiments; triplicates per experiment). ∗*p* < 0.02. *D*, representative immunoblots of total and cleaved (cl) caspase (casp) three and PARP in K562 and HEK293 cells after 48 h of WT NKLAM or C301A expression. Non-induced controls are treated with an equal volume of DMSO (1 μl/ml). Actin serves as a loading control.

K562 cells were induced with Dox to express NKLAM over a 3-day period and analyzed daily by flow cytometry to quantify annexin-V and Ghost Dye Red 780 expression. Representative flow plots are shown in Figure 4*A*. Annexin-V staining is an indicator of apoptosis activation, while staining with Ghost Dye Red 780 signifies loss of membrane integrity. Cells were induced to express either WT NKLAM or C301A, and cultures were analyzed after 24, 48, and 72 h. Over the course of the assay, K562 cells expressing WT NKLAM exhibited an increase in annexin-V and Ghost Dye staining, indicating elevated levels of cell death. In contrast, non-induced controls and cells expressing the ligase deficient C301A variant showed no increase in staining at any time point. The total percentage of cells positive for annexin-V, Ghost Dye, or both, was averaged from 3 separate experiments (Fig. 4*B*). Total annexin-V and Ghost Dye 780 staining increased in cells expressing WT NKLAM to 20% ± 5.5% and 27% ± 2.3% at 48 and 72 h, respectively, while control and C301A-expressing cells remained unchanged during this time course (5.6% ± 1.2%).

The increase in the number of annexin-V and Ghost Dye positive cells in K562 cultures expressing WT NKLAM prompted an investigation into another indicator of apoptosis, caspase activation. A Caspase-Glo 3/7 luminescence assay was conducted with cells induced with Dox for 24, 48, and 72 h to quantitate the activity of caspases three and 7 (Fig. 4*C*). To ensure the integrity of assay reagents and consistent sensitivity, K562 cells were treated with the apoptosis activator staurosporine (stauro) 24 h before each assay, serving as a positive control. WT NKLAM-expressing cells displayed no caspase activity after 24 h. However, at 48 and 72 h, WT NKLAM expression induced increasing caspase activity. Consistent with the flow cytometry analyses, C301A-expressing cells showed no caspase activity at any time point. This result was further corroborated by immunoblot analyses, where cells induced for 48 h revealed the presence of cleaved caspase 3 (cl-casp3) in WT NKLAM-expressing cells. Another marker of apoptosis, cleaved poly (ADP-ribose) polymerase (PARP), was found exclusively in WT NKLAM-expressing K562 and HEK293 cells, while absent in C301A-expressing cells (Fig. 4*D*).

### The NKLAM C301S mutant exhibits ligase-independent activation of apoptosis

**Figure 5:**
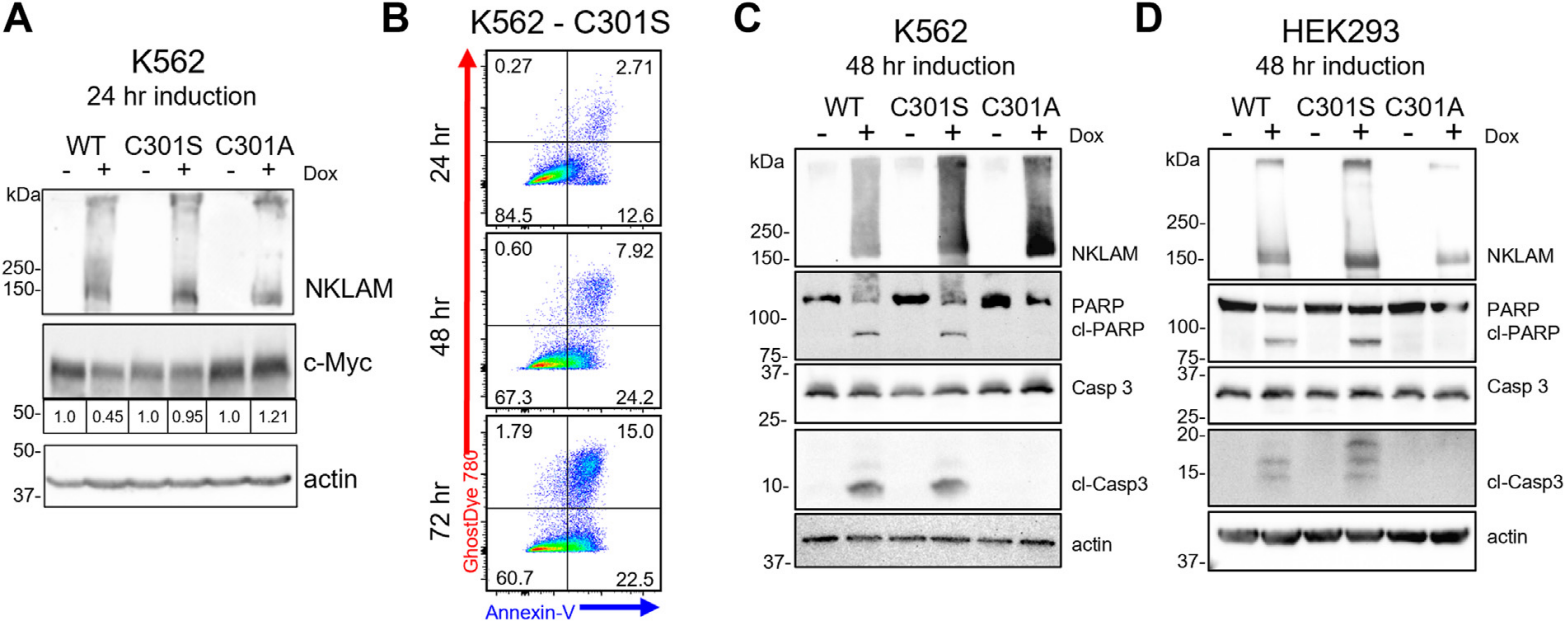
**Ligase-deficient catalytic cysteine to serine mutant of NKLAM activates apoptosis.***A*, representative immunoblot of NKLAM and c-Myc in K562 cells expressing NKLAM WT, C301S and C301A 24 h after Dox induction. Densitometry analyses reflect the levels of c-Myc relative to non-induced controls (−) with actin serving as a loading control. *B*, representative flow cytometry scatter plots of annexin-V and Ghost Dye 780 levels in NKLAM C301S-expressing K562 cells 24, 48 and 72 h after Dox induction. Immunoblots of cleaved caspase 3 (cl-Casp3) and PARP (cl-PARP) in (*C*) K562 and (*D*) HEK293 cells after 48 h of NKLAM, C301S, or C301A expression. Non-induced controls (−) are treated with an equal volume of DMSO (1 μl/ml). Actin serves as a loading control.

K562 cells were induced to express NKLAM, C301S, or C301A for 24 h. Only WT NKLAM expressing cells showed a decrease in c-Myc abundance; neither mutant affected c-Myc expression (Fig. 5*A*). However, by flow cytometry analysis, C301S-expressing K562 cells showed signs of annexin-V and Ghost Dye staining identical to WT NKLAM-expressing cells over the course of 72 h (Fig. 5*B*). K562 and HEK293 cells that expressed the C301S mutant form of NKLAM also displayed signs of apoptosis by immunoblotting, with significant caspase-3 and PARP cleavage (Fig. 5, *C* and *D*). Growth curves for K562 expressing C301S revealed fewer live cells over time compared to controls, but no decrease in c-Myc half-life in a CHX assay (Fig. S3). This suggests that the fewer numbers of C301S-expressing K562 cells over time is due to cell death, not loss of c-Myc activity. These data indicate that the expression of the C301S ligase-dead NKLAM mutant induces apoptosis without decreasing levels of c-Myc.

## Discussion

NKLAM is a membrane-bound E3 ubiquitin ligase important for optimal NK cell effector function. It is localized on lytic granule membranes and extracellular vesicles secreted from these lymphocytes. Our hypothesis is that NKLAM is one of several cytotoxic molecules delivered by NK cells to target cells to induce apoptosis. Here we describe the impact of NKLAM on cellular homeostasis in an ectopic expression model.

RBR ligases like NKLAM utilize a trio of neighboring RING domains to coordinate ubiquitin ligation onto substrate proteins. This is done with a conserved catalytic cysteine on RING2. We first identified this cysteine in NKLAM by sequence alignment with other RBR ligases and here show that mutation of this predicted catalytic cysteine to serine or alanine abrogates NKLAM’s *in vitro* ubiquitin ligase activity. The E2 enzymes UbcH7 and UbcH8 were previously found to interact with NKLAM (29). Here we show that UbcH7 is a functional E2 for NKLAM. UbcH7 is specific for thiol-specific chemistry and therefore can only contribute to ligase activity of E3 enzymes with catalytic cysteines like NKLAM or other RBR ligases (35). This gives us confidence that the activity seen with UbcH7 is specific for NKLAM and unlikely to be background activity of other ubiquitin ligases that may interact with NKLAM. The more broadly active E2 UbcH5c also functions as a Ub donor to NKLAM. NKLAM forms K48 Ub linkages, which are generally regarded as a marker for proteasomal degradation of a substrate protein.

The observed Ub smear starts at the bottom of the membrane at around 25 kDa. This could be due to a small substrate that co-IPs with NKLAM, that is, poly-ubiquitinated in the assay. It could also be caused by a combination of ubiquitination and subsequent de-ubiquitination by a co-purified deubiquitinase enzyme, several of which have been found to interact with NKLAM (36, 37). It may also be due to the generation of unanchored Ub chains. The role of unanchored Ub chains is not well understood but is a growing area of interest (38), and has been shown to be involved in inflammasome activation, leading to cell death.

Interestingly, ubiquitin ligase assays with UbcH8 failed to yield results. The lack of observed ligase activity with UbcH8 could be attributed to its slower enzyme kinetics compared to UbcH7 or to its greater efficiency as an E2 conjugating enzyme for the Ub-like modifier IFN-stimulated gene 15 (ISG15) (39).

This raises an intriguing alternative hypothesis that UbcH8 acts as a donor of ISG15 to NKLAM. ISG15 and the subsequent ISGylation process are known to play critical roles in immune cell function (40), and ISG15 expression in cancer cells has been found to inhibit proliferation and induce apoptosis (41, 42). Other RBR ligases, such as Parkin and Ariadne, exhibit E3 ligase activity for Ub as well as for Ub-like modifiers FAT10 (43) and ISG15 (44), respectively. Therefore, investigating NKLAM’s potential role as an E3 ligase for Ub-like proteins is warranted.

Since NKLAM localizes with other cytolytic proteins in NK cells and its expression is tightly regulated, we hypothesized that NKLAM plays a role in controlling proliferation or inducing apoptosis in target cells. We used an inducible expression system for NKLAM in K562 and HEK293 cells to ensure controlled production of NKLAM. K562 is a myelogenous leukemia cell line and was chosen because it represents a model target for NK cell effector function. HEK293 was used as a representative of an immortalized cell line that is resistant to NK-mediated lysis (45). The expression of NKLAM confirms our hypothesis in both K562 and HEK293 cells that NKLAM is cytotoxic. Induction of NKLAM results in a significant dose-dependent decrease in proliferation. The C301A mutation has no noticeable effect on K562 cell proliferation but does exhibit some impact on HEK293 cells. Upon visual inspection of HEK293 cells expressing C301A, we found alterations in morphology with decreased cellular adhesion, leading to potential erroneous data in our proliferation assays. It is unclear how the catalytically inactive C301A causes this phenotypic change. One hypothesis is that, as a membrane protein, C301A interacts with proteins involved in membrane adhesion or restructuring. The basis of NKLAM C301A’s ability to affect cellular adhesion is currently under investigation.

C-Myc is a transcription factor found primarily in the nucleus of cells and is upregulated in up to 70% of all cancers (46). In normal, non-transformed cells, it plays a pivotal role in the control of metabolism, proliferation, cellular survival, and apoptosis (47). The expression of NKLAM results in a significant decrease in c-Myc abundance. The decrease in c-Myc levels increases over time, indicating that loss of c-Myc is sustained. Additionally, the rescue of c-Myc levels with MG132 indicates that c-Myc continues to be degraded by the proteasome in the presence of NKLAM. Therefore, WT NKLAM-expressing cells produce c-Myc at normal rates, hence the equivalent abundance between induced and non-induced cells after MG132 treatment. From this, we can infer that NKLAM affects c-Myc abundance at the protein level. The CHX assays confirm NKLAM’s effects on c-Myc protein stability by demonstrating the decreased half-life of c-Myc in the presence of NKLAM.

We then tested whether NKLAM promotes the ubiquitination of c-Myc. Multiple methods were employed to address this question, but none provided evidence of increased c-Myc ubiquitination in the presence of NKLAM. We then investigated whether NKLAM promoted the lysosomal degradation of c-Myc by incubating cells with Bafilomycin A1 (BafA1) to block lysosomal activity. Lysosome inhibition failed to rescue c-Myc from degradation (Fig. S4). The results presented in this work unequivocally indicate that NKLAM promotes c-Myc degradation in a proteasome-dependent manner. However, the lack of increased c-Myc ubiquitination by NKLAM suggests that a form of Ub-independent proteasomal degradation may be responsible.

Intrinsically disordered proteins, like c-Myc, can be degraded by the 20S proteasome in the absence of ubiquitination (48, 49, 50). The 20S proteasome complex is not regulated by a 19S subunit that requires a substrate to be ubiquitinated. Proteolytic activity of the 20S proteasome can be further enhanced by the PA28γ subunit. PA28γ is a nuclear-localized alternative to the 19S regulatory subunit that enhances the trypsin-like protease activity of the 20S proteasome. Increasing 20S proteasomal activity, whether by increased expression of the PA28γ proteasomal subunit (51) or by treatment with a small molecule activator like TCH-165 (52), results in a decrease in c-Myc abundance without any evidence of c-Myc ubiquitination, providing a possible explanation of our findings.

Transformed cells often display increased c-Myc expression; this is believed to contribute to higher metabolic activity, uninhibited proliferation, as well as resistance to apoptotic signaling. Numerous studies have shown that silencing of c-Myc by siRNAs can induce apoptosis in a variety of cancer and normal cell lines (53, 54, 55). This is in addition to G1/S phase cycle arrest, decreased metabolic function, and increased sensitivity to pro-apoptotic signaling. WT NKLAM is shown in this study to decrease the abundance and stability of c-Myc. In the context of immune cell effector function, the degradation of c-Myc in the presence of NKLAM may contribute to the control of diseased cells, providing an alternate pathway to preventing metastasis or sensitizing cells to apoptosis.

Loss of c-Myc by NKLAM could lead to apoptosis. However, cells expressing the ligase-inactive C301S mutant do not show loss of c-Myc but still become apoptotic, indicating that the activation of apoptosis is independent of the degradation of c-Myc. There is precedent for the activity of an RBR ligase with a catalytic cysteine-to-serine mutation (56). One study showed that the catalytic cysteine to serine (C431S) mutant of Parkin indirectly promotes ubiquitination and degradation of TNF receptor-associated factor 3 (TRAF3) in a ligase-independent manner. The authors suggested that the interaction between Parkin and TRAF3 promotes ubiquitination of TRAF3 by other nearby E3 ligases or that the interaction inhibits the activity of deubiquitinases.

What distinguishes WT NKLAM and the C301S mutant from C301A is their ability to bind an active Ub at the catalytic site. NKLAM forms a thioester bond with the C-terminal carboxyl group of Ub at the catalytic site. Mutation of the catalytic cysteine to alanine would eliminate binding of Ub, but a serine substitution would result in an ester bond that is resistant to acyl substitution by the lysine of a substrate protein, preventing transfer of Ub. The significance of Ub binding to the catalytic site was demonstrated by crystallographic analysis of another RBR ligase, Ariadne (HHARI or ARIH) (57). In this study, the authors investigated the transition states of Ariadne during ubiquitination and described the arrangement of the RING domains of Ariadne with and without a Ub bound to the catalytic site. They showed that with Ub conjugated to the catalytic site, the RING domains rearrange, moving the catalytic site 60Å closer to the substrate protein to facilitate ligation. This study demonstrates the flexibility and high mobility of the individual RING domains of Ariadne which may also be a factor for other RBR ligases. NKLAM may undergo a similar structural rearrangement when Ub is attached to the catalytic cysteine or trapped on the serine substitution, and this rearrangement of NKLAM’s domains may be responsible for the activation of apoptosis. The C301S mutant allows us to study how NKLAM activates apoptosis separately from its ubiquitin ligase function. Further structural analysis of NKLAM is warranted.

**Figure 6:**
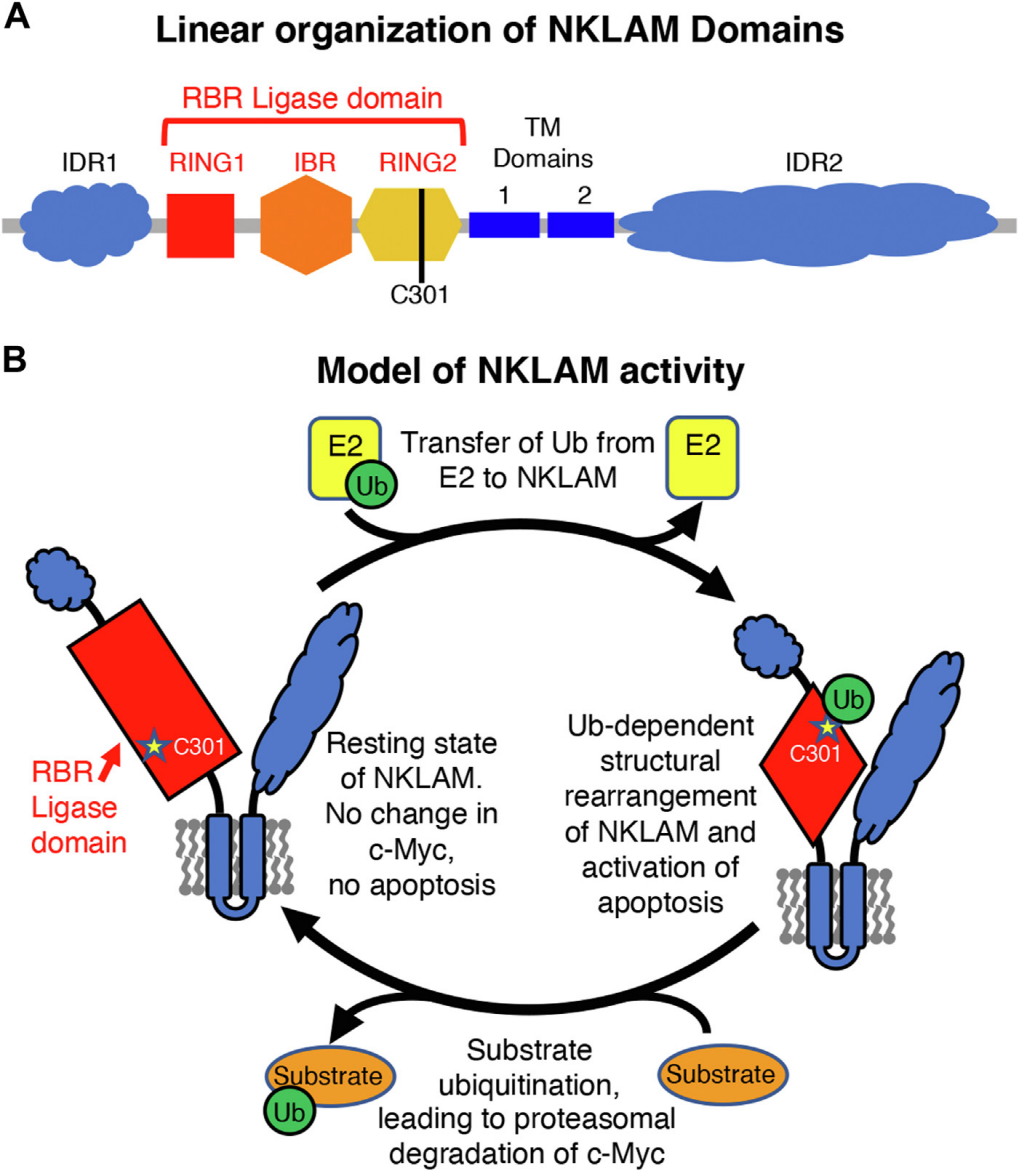
**Proposed mechanism for NKLAM’s dual activity.***A*, linear diagram of NKLAM structural domains and location of the catalytic cysteine (C301). NKLAM contains two transmembrane (TM) domains that position the N- and C-terminal regions on the same side of a membrane. The two intrinsically disordered regions (IDR1/2) have no known function or homology to other proteins. *B*, NKLAM has two independent functions. The pro-apoptotic activity of WT NKLAM and the C301S mutant is dependent on E3-Ub conjugation, which may result in a rearrangement of structural domains to trigger apoptosis. The loss of c-Myc is due to the Ub ligase activity of WT NKLAM and is proteasome dependent.

In conclusion, the expression of WT NKLAM inhibits proliferation, reduces c-Myc levels, and induces apoptosis; the ligase-dead C301A NKLAM mutant has no effect. Expression of the C301S NKLAM mutant in K562 and HEK293 cells activates apoptosis but does not affect c-Myc abundance. This informs us that NKLAM inhibits proliferation and induces apoptosis by two different mechanisms: the former, dependent on Ub ligase activity, and the latter dependent on the binding of Ub to NKLAM’s catalytic site to form an E3-Ub conjugate. A model of the proposed mechanism for NKLAM’s dual activity is shown in Figure 6.

As an RBR E3 ligase, NKLAM represents another class of cytolytic molecules not previously identified as part of NK effector function. Its dual role in the degradation of c-Myc by ubiquitination, and in the ligase-independent activation of apoptosis makes it a potentially potent tool in the arsenal of cytotoxic lymphocytes. Tumor cells may mutate or transform in a way that confers resistance to perforins, granzymes, death receptor signaling, or some combination (58, 59, 60). The inclusion of NKLAM in the cytotoxic process presents yet another obstacle for the tumor cell to develop a strategy to evade.

## Methods

### Cell culture

HEK293 cells (ATCC) were maintained in a sterile humidified culture chamber at 37 °C with 5% CO_2_ in Dulbecco's Modified Eagle Medium (DMEM) supplemented with 10% fetal bovine serum (FBS) and 1% penicillin-streptomycin solution. Cells were routinely passaged every 3 to 5 days to maintain exponential growth. Upon reaching approximately 80 to 90% confluency, cells were detached using a 0.25% trypsin-EDTA solution and resuspended in complete growth medium. The cell suspension was then counted, diluted to the desired cell density for seeding into 10 cm culture plates or multi-well plates as needed for experimentation. MG132 (10 μM final concentration) or Bafilomycin A1 (BafA1) (100 nM final concentration) were added to cells in culture media for 3 h prior to harvest.

K562 cells (ATCC, Manassas, VA) were maintained in a sterile humidified culture chamber at 37 °C with 5% CO_2_ in RPMI 1640 medium supplemented with 7.5% FBS and 1% penicillin-streptomycin solution. Every 2 to 3 days, the cells were counted and diluted into new T25 or T75 flasks with fresh culture medium.

For all cultures, regular microscopic examination was conducted to monitor cell morphology and contamination. Additionally, *mycoplasma* testing was performed periodically to ensure the absence of microbial contamination.

### Plasmid design

The PCS2+MT vector was utilized for the mutagenesis of NKLAM and attachment of fluorescent probes and affinity tags. The multicloning site (MCS) of the vector was modified to alter the restriction sites available and remove the myc tag that was present. NKLAM was amplified out of an existing plasmid construct and subsequently ligated into the modified PCS2 vector. The forward primer: 5′-ACTGACACGCGTCCACCATGGGCTCCGAGAAGGAC-3′ and reverse primer: 5′-ACTGACACCGGTACTCTGGCTTCTCCACCTTC-3′ provided restriction sites (MluI and AgeI at the 5′ and 3′ ends, respectively) to the amplified NKLAM product for ligation into the new MCS. Amplification was performed using the Phusion High Fidelity DNA Polymerase with HF buffer (New England Biolabs; Cat# M0531), with a Mastercycler EP gradient thermocycler (Eppendorf model 5340). The NKLAM insert was sequence verified to ensure fidelity of the amplification before mutagenesis. NKLAM constructs were modified to express a C-terminal mScarlet fluorescent probe and a V5 affinity tag for IPs unless otherwise stated. An identical MCS was also placed into the Sleeping Beauty plasmid, a gift from Eric Kowarz (Addgene #60501) (27). Transposase plasmid pCMV(CAT)T7-SB100 was also a gift from Eric Kowarz (Addgene #34879) (28).

### Site-directed mutagenesis

Site-directed mutagenesis was used to generate C301S and C301A mutations to NKLAM with the Agilent Quickchange II kit (Agilent; P/N: 200,524) and Mastercycler EP gradient thermocycler. Reactions were done according to the manufacturer’s protocol but supplemented with 2.5% DMSO and 2.5% ethylene glycol (v/v final concentration) to ensure efficient polymerization through NKLAM’s GC-rich sequence. Table 1 lists the primers used for mutagenesis of NKLAM’s catalytic cysteine. They were carefully designed to ensure specificity to the target sequence, but also to prevent secondary structure while also maintaining matching melting points for primer pairs. Mutant constructs were sequence verified.

**Table 1:** Primers for site-directed mutagenesis

Primer name	Sequence
NKLAM C301A-F	ATGAATGATGGAAGCGCTAATCACATGACCT
NKLAM C301A-R	AGGTCATGTGATTAGCGCTTCCATCATTCAT
NKLAM C301S-F	ATGAATGATGGAAGCTCTAATCACATGACCT
NKLAM C301S-R	AGGTCATGTGATTAGAGCTTCCATCATTCAT

### Inducible cell line generation

K562 and HEK293 cells were modified to contain a construct with a tetracycline/doxycycline (Dox)-inducible promoter to control NKLAM protein expression. This was accomplished with the Sleeping Beauty transposon containing green fluorescence protein (GFP) and G418 resistance for antibiotic selection (28). The transposon plasmid containing the NKLAM-mScarlet-V5 construct, and a separate transposase plasmid were nucleofected or transfected into K562 and HEK293 cells, respectively. Nucleofection of K562 cells was done with the Amaxa nucleofector II (Lonza) in combination with Kit V (Lonza, VCA-1003), using program X-005. Cells were cultured for 24 h in standard growth media before selection with G418. After 2 weeks of selection, cells were then subjected to cell sorting with a FACSAria III (BD Biosciences) to select GFP-positive cells. Cells were sorted and selected for comparable expression of their respective NKLAM constructs.

Transfection of HEK293 cells was accomplished with the Lipofectamine 3000 Transfection Reagent kit (ThermoFisher; cat#: L3000001). The Sleeping Beauty plasmid containing the NKLAM construct was co-transfected into cells with the Sleeping Beauty transposase plasmid. After overnight incubation, the media was replaced and G418 selection started. After 2 weeks of selection, cultures for each construct (WT, C301S, and C301A) were put through two rounds of single cell expansions and selected for comparable expression of their respective NKLAM constructs.

### Ubiquitin ligase assays

HEK293 cells were cultured overnight in 10 cm culture plates with 1 μg/ml Dox to induce expression of NKLAM or mutant variants, or with an equivalent volume of DMSO as a negative control treatment. Cells were harvested and lysed in lysis buffer (LB#2) containing 65 mM Tris pH7.5, 137 mM NaCl, 10% glycerol, 1% Triton X-100. LB#2 was supplemented with Pierce protease inhibitor cocktail, EDTA free (ThermoFisher cat#: A32963). Lysis was followed by a high-speed spin to pellet insoluble material, and the supernatants were transferred to new tube. Solubilized NKLAM in the supernatant was IP’d onto protein G beads (Millipore Sigma; Cat#: GE17–0618–01) using an anti-V5 antibody (Invitrogen; Cat#: MA5-15253) overnight. The beads were subsequently washed with LB#2 3 times, then buffer exchanged into ligase reaction buffer containing 50 mM Tris pH 8.0, 50 mM NaCl, 10 mM MgCl_2_, 0.2% NP-40, 1 mM DTT. Beads were split as needed for control and test reactions. 100 nM E1 (R&D systems Cat#: E304–50), 1 μM E2 (R&D systems UbcH5c Cat# E2-627, UbcH7 Cat#: E2-640, UbcH8 Cat#: E2-643), 25 μM HA-tagged ubiquitin (R&D systems Cat#: U-110–01M), and 10 mM ATP (Sigma cat#: A9187) were added to the individual reactions. Reactions were performed in a shaking incubator at 37 °C for 3 h, then quenched by addition of SDS-PAGE sample buffer containing 5% v/v β-mercaptoethanol (BME), 10% glycerol, 100 mM Tris pH 6.8, 2% SDS w/v, and bromophenol blue. Samples were denatured at 95 °C for 5 min. Reactions were analyzed by immunoblotting.

### Proliferation assays

K562 cells were placed in fresh growth media 24 h prior to experimental setup. On the day of setup, cells were counted and seeded into 6-well plates with 1 μl/ml DMSO, or 1 μg/ml Dox. At 24-h intervals, cells were collected, and 10 μl of each culture mixed 1:1 with trypan blue solution and counted using a Countess II FL (Invitrogen). Remaining cells had media and DMSO or Dox refreshed, and cells placed back into new plates.

For HEK293 proliferation assays, cells were trypsinized, washed in media, and counted. An identical number of cells were seeded into multi-well plates and allowed to adhere overnight. One well was seeded for each designated timepoint per condition. The following day, cells were treated with either 1 μl/ml DMSO, or 1 μg/ml Dox. At 24 h intervals, one well for each condition was trypsinized, washed, and resuspended in 10 to 500 μl depending on the expected cell count to ensure appropriate density and accuracy of the count. Different well plate sizes were used for different time points to prevent cells from reaching confluency during the assay.

### WST-1 assays

K562 stock cultures were fed 24 h prior to experimental setup. On day of setup, cells were counted and 5000 cells seeded in 96-well plates with 1 μl/ml DMSO, or 1 μg/ml Dox. At 24-h intervals, cells were transferred to a secondary plate, mixed with 10 μl WST-1 reagent and placed back in the incubator for 2 h. Absorbance at 450 nm was measured using a BioTek Gen five multi-well microplate reader.

### Crystal violet assays

HEK293 cells were seeded into 96-well plates at a density of 2000 cells per well and allowed to adhere overnight. The following morning, the media was refreshed and supplemented with Dox or an equal volume of DMSO for controls. At time zero and at every 24 h interval, wells were aspirated to remove media, washed once with PBS, and treated with crystal violet staining solution for 10 min. The wells were washed with PBS again, then treated with 10% acetic acid for 10 min. Absorbance at 570 nm was measured using a BioTek Gen five multi-well microplate reader.

### Caspase Glo-3/7 luminescence assays

K562 cells were harvested during their exponential growth phase and seeded into 12-well plates at a density of 5 × 10^4^ cells per well with either Dox or an equal volume of DMSO. At 24, 48 and 72 h after plating, a well for each condition was collected, resuspended in fresh media, and counted. Five thousand live cells from each condition were added to three wells in a 384-well plate in 10 μl. 10 microliters of Caspase-Glo 3/7 reagent (Promega; Cat#: G8090) was added to each well, plates were incubated for 30 min, then luminescence was measured in a BioTek Gen five plate reader (BioTek).

### Flow cytometry and cell sorting analyses

After nucleofection with the Sleeping Beauty transposon and transposase plasmids, K562 cells were cultured in G418 for 2 weeks, then sorted twice based on GFP expression, using either a FACSAria Fusion or FACSAria III (BD Biosciences).

For flow cytometry experiments, cells were harvested and washed with PBS, then resuspended in annexin binding buffer containing 10 mM HEPES pH 7.4, 140 mM NaCl, and 2.5 mM CaCl_2_. Cells were then stained with eFluor 450-annexin-V (BioLegend, Cat#: 640908), and Ghost Dye 780 (Cytek; Cat#:13–0865-T100) for 15 min, then diluted with additional annexin binding buffer prior to analysis in an LSRII flow cytometer (BD Biosciences). Cells were also monitored for expression of GFP and mScarlet. The gating strategy is described in Figure S5. Data were analyzed with FlowJo software.

### Immunoblotting

Whole cell lysates were prepared with either LB#2 or RIPA lysis buffer, containing protease inhibitors (ThermoFisher cat#: A32963). Lysates were spun down to remove insoluble materials, supernatants were mixed with Laemmli sample buffer denatured at 95 °C for 5 minutes, separated by SDS-PAGE, and transferred onto polyvinylidene fluoride (PVDF) membranes by semi-dry transfer. Membranes were blocked with 5% BSA in Tris-buffered saline (TBS), with 0.5% Tween 20 (TBST) for 10 min and incubated overnight at 4 °C in primary antibody diluted in blocking buffer. Membranes were washed 3 times in TBST and incubated in TBST containing horseradish peroxidase (HRP)-coupled secondary antibody on a rocker for 1 h at room temperature. Membranes were washed again 3 times with TBST, and proteins were detected using either Clarity ECL (Bio-Rad) or WesternBright enhanced chemiluminescence (ECL) HRP substrate (Advansta) and a Chemi-Doc imaging system (Bio-Rad). Densitometry analyses of immunoblots were performed with Image Lab v 6.0.1 software (Bio-Rad). Antibodies to β-actin (Sigma; A5441), V5 (Invitrogen; MA5-15253 and Cell Signaling; 13202S), c-Myc (Cell Signaling; 5605S), K48 Ub (Cell Signaling; 8081S), K63 Ub (Cell Signaling; 5621S), HA (ThermoScientific; 26,183), GAPDH (Cell Signaling; 2118S), caspase 3 (Cell Signaling; 14220S), cleaved caspase 3 (Cell Signaling; 9664T), PARP (Cell Signaling; 9542S), cleaved PARP (Cell Signaling; 5625T).

### Cycloheximide (CHX) assays

K562 cells were cultured in growth media in 6-well plates and treated with 1 μg/ml Dox, or 1 μl/ml DMSO overnight in a humidified incubator at 37 °C with 5% CO_2_. The following day, the cultures were pelleted and resuspended in 3 ml of culture media containing 100 μg/ml CHX, and aliquoted into separate six wells of a 24 well plate and returned to the incubator. At designated time points, one well was harvested for each condition, cells pelleted in a pre-chilled centrifuge, immediately lysed in ice-cold radioimmunoprecipitation assay (RIPA) buffer containing protease inhibitors and 100 mM n-Ethylmaleimide and denatured in SDS-PAGE buffer containing 5% v/v BME. Samples were analyzed by immunoblotting. Data from three or more assays were analyzed by regression analysis.

### Immunoprecipitations (IPs)

NKLAM or c-Myc were IP’d from lysates using anti-V5 (Invitrogen; Cat#: MA5-15253), or anti-c-Myc (Santa Cruz; Cat#: sc-42) antibodies, respectively, at 1 μg per IP with 10 μl protein G beads (Millipore Sigma; Cat#: GE17–0618–01). For NKLAM IPs, cells were lysed in LB#2 with protease inhibitors, insoluble material spun out, and supernatants moved to new tubes where antibody and protein G beads were added. NKLAM IPs were carried out overnight with rocking at 4 °C. For c-Myc IPs, cells were first lysed in LB#2 with protease inhibitors, insoluble material pelleted, and supernatants removed. The insoluble pellets containing intact nuclei were lysed with RIPA buffer supplemented with protease inhibitors, and insoluble material spun out again. Supernatants were moved to a new tube where antibody and protein G beads were added. c-Myc IPs were incubated for 1 h, rocking, at 4 °C. After incubation, beads were then spun down, supernatants removed, washed 3 times in lysis buffer and prepared in SDS-PAGE sample buffer.

### TUBEs pull down assays

Tandem ubiquitin binding entities (TUBEs) are a fusion of multiple Ub interacting domains capable of binding ubiquitinated proteins (34). The TUBE construct (pET pET28a-6HIS_TEV-HALO-ubiquilin TUBE), obtained from the MRC PPU Unit at the University of Dundee, was grown in bacteria and the TUBEs isolated using HaloTag beads (Promega). Cell lysate samples were normalized by protein concentration and bead-bound TUBEs added to lysates and incubated at 4 °C overnight with rocking. The beads were then collected by centrifugation, washed 3 times with RIPA and prepared in SDS-PAGE sample buffer for immunoblotting.

### Statistics

Statistical analyses were performed with two-tailed unpaired Student’s *t*-tests to compare two groups. Regression analysis was used for CHX assay results. All statistical analyses were done using Microsoft Excel.

## Data availability

All data are contained within the manuscript.

## Supporting information

This article contains supporting information.

## Conflict of interest

The authors declare that they have no conflicts of interest with the contents of this article.
