# Supplementary material for: The ubiquitin ligase NKLAM promotes apoptosis and suppression of cell growth
Source: J Biol Chem. 2025 Apr 22;301(6):108527. doi: 10.1016/j.jbc.2025.108527 (PMC12148440; doi:10.1016/j.jbc.2025.108527)
Supplement: Supporting Figures [file mmc1.docx]

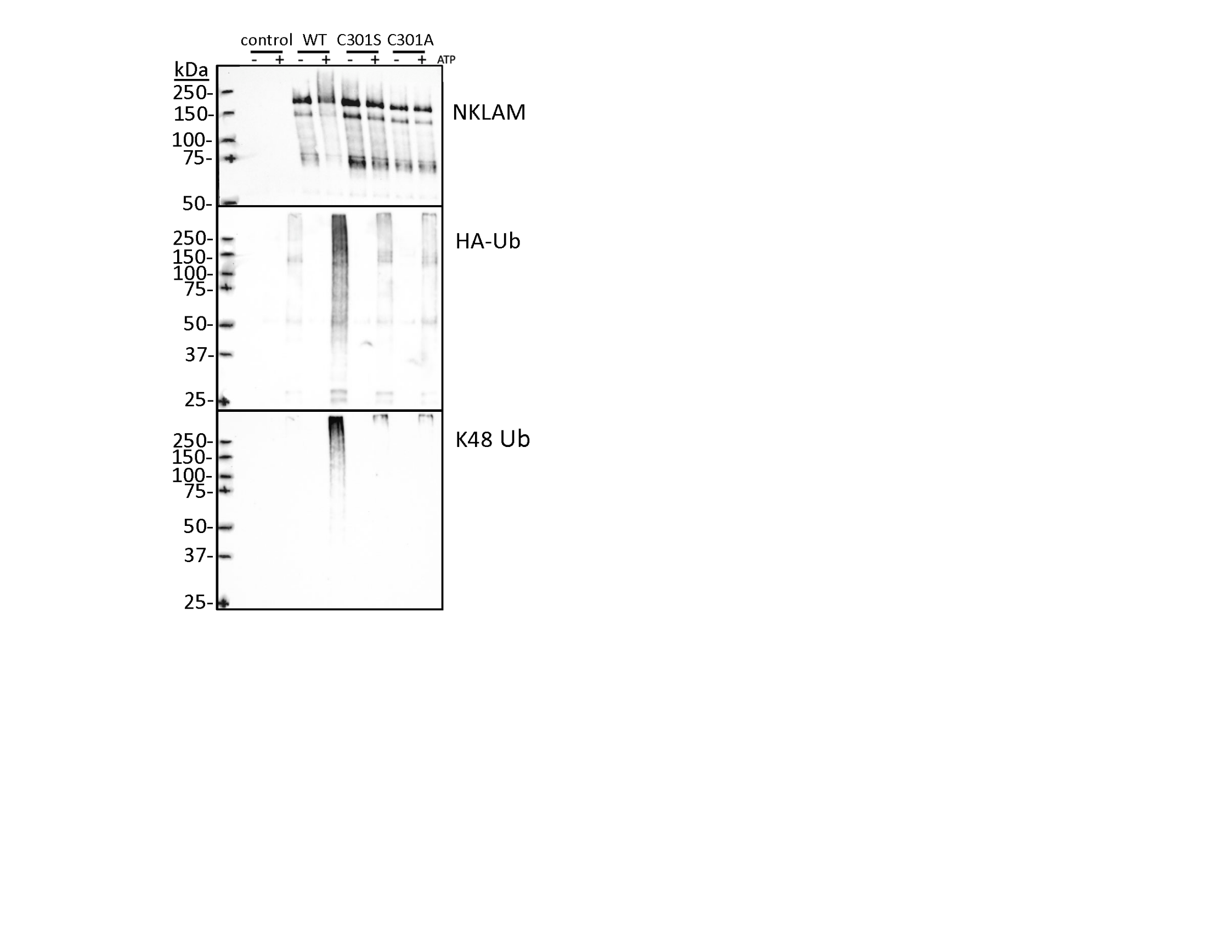


Supporting Figure 1. NKLAM forms K48 Ub linkages.

Representative immunoblot of an *in vitro* ubiquitin ligase assay using NKLAM WT, C301S and C301A proteins IP’d from HEK293 whole cell lysates and incubated with E1, E2 (UbcH7), HA-Ub +/- ATP. WT NKLAM but not the catalytic cysteine mutants display significant Ub ligase activity; K48 Ub linkages are seen in proteins IP’d with WT NKLAM.


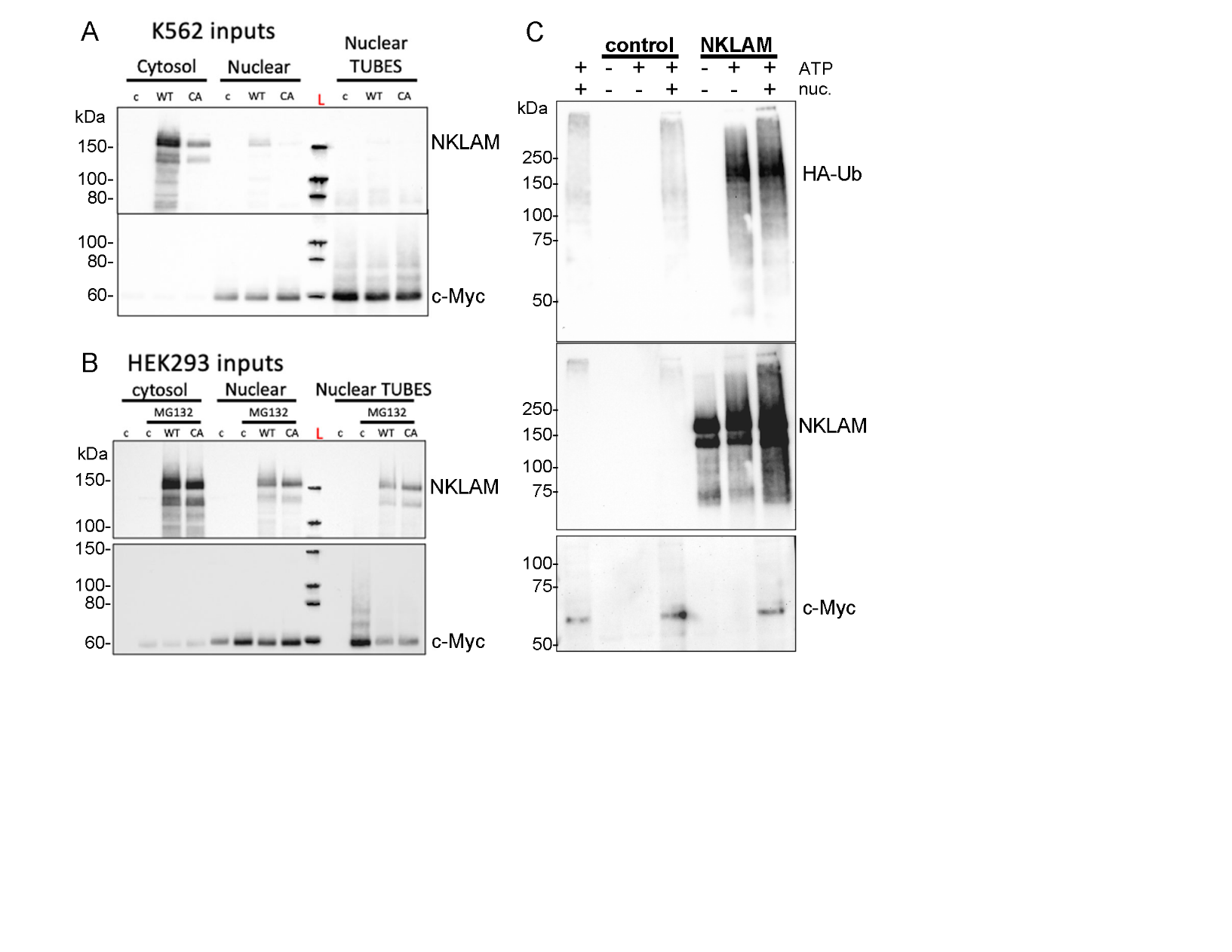


Supporting Figure 2. NKLAM does not ubiquitinate c-Myc.

(A) Ubiquitinated proteins in nuclear fractions of lysates from MG132-treated K562 cells using TUBEs. Immunoblot for c-Myc reveals no change in c-Myc ubiquitination in WT NKLAM expressing cells compared to control or C301A-expressing cells. (B) Immunoblot of ubiquitinated proteins in nuclear material isolated from HEK293 cells using TUBEs. There is less ubiquitination of c-Myc in cells expressing WT NKLAM or C301A. (C) Immunoblot of a representative *in vitro* ubiquitin ligase assay using nuclear fractionated material from non-induced HEK293 cells. NKLAM is IP’d with antibody onto protein G beads from whole cell lysates, and incubated with E1, E2, HA-Ub, +/-ATP, and enriched nuclear material. c-Myc reveals no apparent change in ubiquitination. NKLAM shows enhanced ubiquitination with the addition of nuclear lysate, and ubiquitin ligase activity in the form of increased HA-Ub smearing.


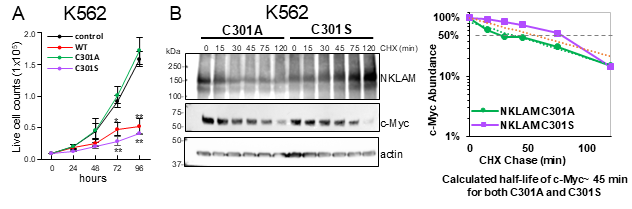


Supporting Figure 3. Expression of the NKLAM C301S mutant inhibits proliferation but does not affect c-Myc abundance or half-life.

(A) Growth curve of K562 cells expressing C301S plotted with growth curves shown in Figure 2A. Both WT NKLAM and C301S-expressing cells show less proliferation than control or C301A-expressing cells. **p* < 0.05, ***p* < 0.0005. (B) CHX assay comparing the levels of c-Myc in NKLAM C301A and C301S expressing K562 cells. c-Myc abundance decreases at identical rates and the calculated half-lives are nearly identical at 46 ± 4.4 minutes.


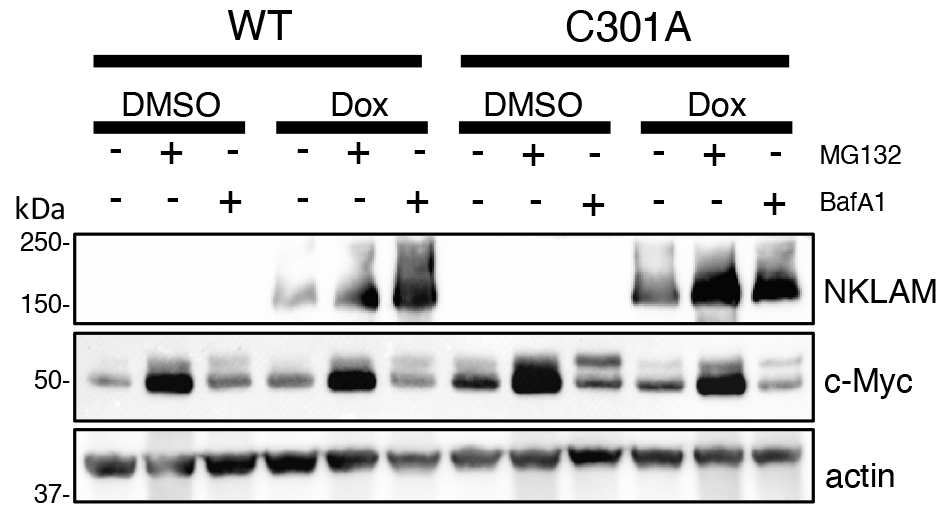


Supporting Figure 4. Inhibition of lysosomal degradation with BafA1 does not rescue c-Myc.

Representative immunoblot of c-Myc and NKLAM in K562 cells induced to express WT NKLAM or C301A for 24 hours, followed by a 3 hour treatment with MG132 or BafA1. Treatment of controls and induced cells with MG132 rescues c-Myc from proteasomal degradation, resulting in denser bands. However, BafA1 treatment does not rescue c-Myc, indicating that NKLAM does not promote lysosomal degradation of c-Myc. Both MG132 and BafA1 increase expression of WT NKLAM and C301A, suggesting that a portion of NKLAM is degraded by the lysosome and some by the proteasome.


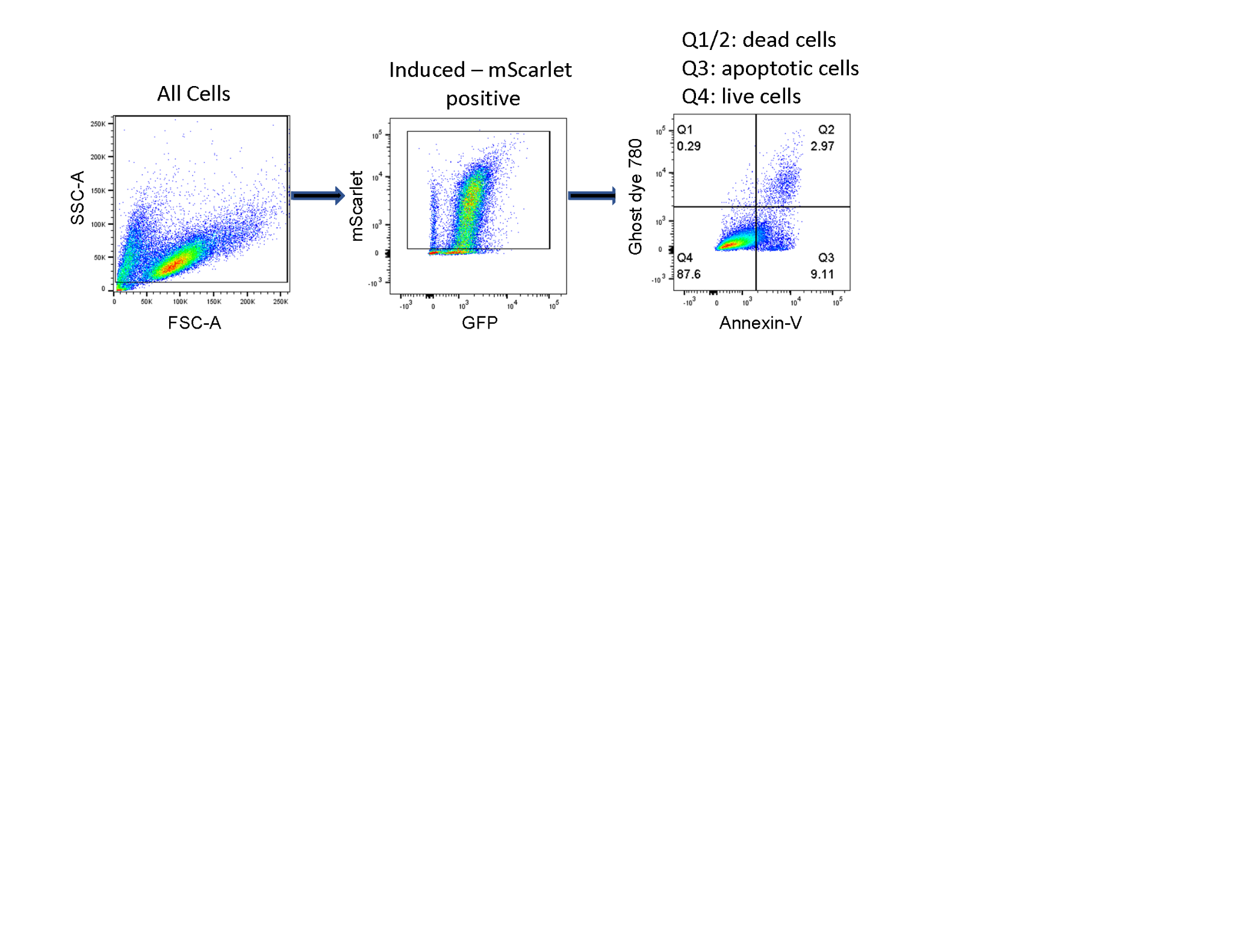


Supporting Figure 5. Flow analysis gating strategy.

Live and dead cells are selected, excluding cell debris (first plot). The fluorescent protein mScarlet is attached to NKLAM, so mScarlet-positive cells (y-axis in the second plot) are selected for analysis of annexin-V (y-axis) and Ghost Dye 780 (x-axis) staining (third plot).
